# A survey on the current status of *Helicobacter pylori* infection in households in Hainan Province, China

**DOI:** 10.1186/s12876-023-03010-z

**Published:** 2023-12-04

**Authors:** Danni Liu, Jing Pan, Zhengyi Chen, Sailian Li, Jiamei Ma, Yening Xiao, Danhong Wang, Ganggang Mu, Ya Lin, Juyuan Li, Zhai Chen, Xiaoxi Huang

**Affiliations:** 1 Department of Gastroenterology, Central South University Xiangya School of Medicine Affiliated Haikou Hospital, Haikou, China; 2 Department of Pediatrics, Central South University Xiangya School of Medicine Affiliated Haikou Hospital, Haikou, China; 3 Department of Gastroenterology, Hospital of Wuhan University, Wuhan, China; 4 Department of Gastroenterology, Tongji Wenchang Hospital of Huazhong University of Science and Technology, Wenchang, China; 5 Department of Gastroenterology, Hainan West Central Hospital, Danzhou, China; 6 Digestive Endoscopy Center, Dongfang People’s Hospital, Dongfang, China

**Keywords:** Helicobacter pylori, Survey, Questionnaire, Households, Prevalence

## Abstract

**Objective:**

This study aims to assess the prevalence of *Helicobacter pylori* (*Hp*) infection at the household level in Hainan Province in China and identify the factors that contribute to its spread. The findings of this study have significant implications for public health prevention strategies in the Hainan region.

**Methods:**

A total of 421 families, comprising 1355 individuals, were tested for *Hp* infection across five cities in Hainan Province between July 2021 and April 2022. The study utilized questionnaires that included questions about personal characteristics, household shared lifestyle and dietary habits, and potential pathways of Hp infection in children to identify potential factors linked to household *Hp* infection and transmission patterns.

**Results:**

The prevalence of *Hp* infection on an individual basis was 46.72% (629/1355), with age ≥ 20 years, being married and having junior secondary education and above as risk factors for *Hp* infection. The prevalence of *Hp* infection in households was 80.29% (338/421), household size of 5, 6 and above were risk factors for *Hp* infection with Odds Ratios (ORs) of 4.09 (1.17–14.33) and 15.19 (2.01–114.73), respectively, household income ≥ 100,000 yuan and drinking boiled water from a tap source were protective factors for *Hp* infection with ORs of 0.52 (0.31–0.89) and 0.51 (0.28–0.95), respectively. The prevalence of *Hp* infection among minors in the household was 24.89% (58/233), with paternal infection and maternal infection as risk factors for child infection, with ORs of 2.93 (1.29–6.62) and 2.51 (1.07–5.89), respectively.

**Conclusion:**

*Hp* infection was prevalent among Hainan families, and interaction with infected family members may be the primary cause of transmission.

**Supplementary Information:**

The online version contains supplementary material available at 10.1186/s12876-023-03010-z.

## Introduction

*Helicobacter pylori* (*Hp*) is a gram-negative bacterium that colonizes the gastric mucosa and is clearly certified as a class I carcinogen for gastric cancer [1]. In 2015, there were approximately 4.4 billion persons worldwide infected with *Hp*, with the rate of infection in China ranging from 20.6% to 81.8% due to the enormous territory and varied levels of development in different regions [2, 3]. *Hp* is transmitted from person to person, particularly between family members, and an infected family member can be a serious source of infection, putting other family members at risk [4]. Hainan Province is located in the southernmost part of China and only surveys of *Hp* infection in specific populations are available. There are no large-scale surveys of household *Hp* infection status in the population of Hainan Province. Moreover, the patterns of intra-household transmission of *Hp* and factors associated with pathogenicity are not known. The aim of this study is to evaluate the prevalence of *Hp* infection, related risk factors, and possible channels of transmission in the average household in Hainan Province. The findings will contribute to gather evidence on the familial aggregation of *Hp* infection in the Hainan region.

## Methods and materials

### Study population

Based on a 50% *Hp* prevalence rate in China and a relative error of 5% (α = 0.05), the required sample size was calculated to be 1536. The study was conducted from July 2021 to April 2022 in five prefecture-level cities in Hainan Province, specifically Haikou, Danzhou, Dongfang, Wenchang, and Changjiang Li Autonomous County, where study participants were recruited. A total of 1,454 people participated in the questionnaire and test, with a response rate of 94.7%. Based on the results of the questionnaire, 421 households (1355 persons) were finally screened out after excluding unqualified questionnaires. The study conducted by the researchers excluded certain sensitive groups such as pregnant women and breastfeeding mothers, as well as individuals taking proton pump inhibitors, antibiotics, bismuth or herbs with antibacterial effects within a month. Additionally, households with only one resident were also excluded from the study. Participants were recruited based on their actual co-residence, rather than their household registration. Co-residence was defined as living together for more than 10 months per year over the last 5 years, as determined by at least 2 permanent co-residents. All subjects in the study completed questionnaires and were tested for *Hp*.

### Questionnaire

With the assistance of uniformly trained researchers, the questionnaire was completed by all participants or their guardians through scanning a QR code to access a small app on the WeChat platform. The researchers carefully collected data and excluded any invalid questionnaires. The questionnaire consisted of questions regarding personal characteristics, lifestyle and dietary habits, potential routes of *Hp* infection in children within the family, et al.

### Testing for *Helicobacter pylori*

The ^13^C-Urea Breath Test (^13^C-UBT) is the most extensively researched and highly recommended non-invasive method for detecting *Hp* infection [5]. In this study, participants including adults, adolescents, and select children underwent the ^13^C-UBT using a uniform kit (Shenzhen Zhonghe Headway Bio-Sci & Tech Co., Ltd, Shenzhen, China). The participants were instructed to fast for at least two hours prior to the test in the morning. The first respiratory sample, labeled as sample 1, was collected in a blue exhalation collection bag. Following this, participants were instructed to take a urea ^13^C capsule with 80-100ml of drinking water and remain seated for 30 min. Afterwards, they were asked to exhale again, and this second sample was collected in a green exhalation collection bag labeled as sample 2. Testers analyzed samples 1 and 2 by using the ^13^C breath detector (HCBT-01, Shenzhen Zhonghe Headway Bio-Sci & Tech Co., Ltd, Shenzhen, China). If the delta over baseline value ≥ 4.0, the result was positive for *Hp* infection. The stool antigen test (SAT) is an alternative detection method with high specificity and sensitivity [6]. As part of the study, children primarily under the age of 4 or those unable to cooperate in the ^13^C-UBT underwent SAT. Fresh fecal samples were collected from participants and tested using the *Hp* antigen test kit (Huagen tailai Biotechnology Co., Ltd, Jiangsu, China). In a clinical trial in Xiamen, China, the sensitivity of the SAT reached 93.8% and the specificity reached 96.6% [7]. A positive result for *Hp* is indicated by a red T line on the test. If at least one member of a family is infected with *Hp*, the entire family is considered *Hp*-infected. Conversely, if no members of a family are infected with *Hp*, the family is considered *Hp*-uninfected.

### *Statistical* analysis

This study used SPSS statistical software version 26.0 (IBM Corporation, Armonk, NY, USA) to analyze data. The count data was presented using frequencies and rates (%), and statistical comparisons between groups were conducted using the chi-square test, Fisher's exact probability method, and the continuity correction test. The measurement data was presented as mean ± standard deviation, and group comparisons were conducted using t-tests. Statistical significance was determined at a bilateral *p*-value < 0.05. In this study, logistic regression analysis was utilized to evaluate the potential factors affecting *Hp* transmission. The findings were reported as an Odds Ratio (OR) along with its corresponding 95% Confidence Interval (CI).

## Result

### *Helicobacter pylori* infection in individuals

**Fig. 1:**
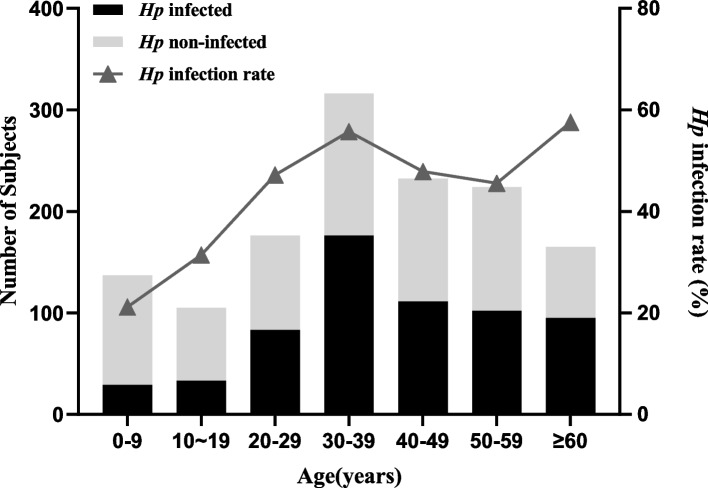
*Helicobacter pylori* infection status in patients of different age groups Note: *Hp Helicobacter pylori *

A total of 1355 individuals were tested for *Hp* infection, with 629 (46.42%) testing positive and 726 (53.58%) testing negative. The prevalence rate varied by age group, with the lowest rate of 21.17% (29/137) found in the 0–9 years age group and the highest rate of 57.58% (95/165) found in the ≥ 60 years age group. The prevalence of *Hp* infection generally increased with age, particularly in the 0–40 years age group, and decreased in the 40–60 years age group. However, it reached its highest point in individuals aged 60 years or older (Fig. 1).

**Fig. 2:**
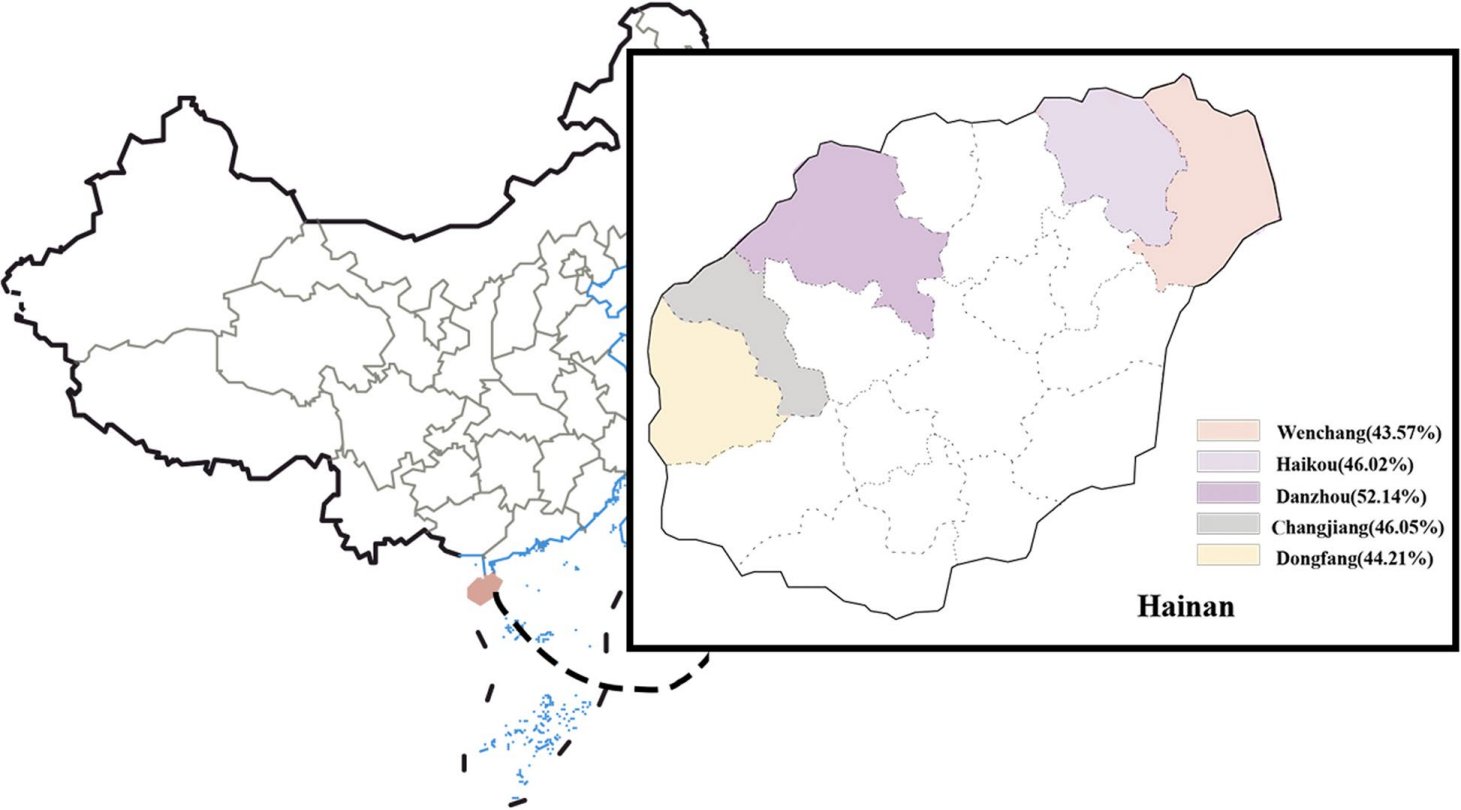
Distribution of *Helicobacter pylori* positive infection rate in each involved municipal area in Hainan

In a univariate analysis, we screened for significant effects of age, education level, and marital status (*p* < 0.05) on an individual's *Hp* infection status. Subsequently, a multifactorial analysis revealed that only age had a greater effect on *Hp* infection in the population. With the age range of 0–9 years as the reference, the risk of *Hp* infection was higher in those aged 20 years and older, with the highest risk of infection in those aged 60 years and older with an OR of 4.01 (1.83–8.79). Considering that the population included in this study was all-age, and that age actually had a greater influence on marriage and education level, we excluded the age factor and found that married, middle school and education above high school and college were risk factors for *Hp* infection, with ORs of 1.64 (1.27–2.13), 1.64 (1.18–2.29) and 1.65 (1.22- 2.22) (Table 1). Of the five prefecture-level cities in Hainan Province, Danzhou had the highest rate of *Hp* infection at 52.14%, while Wenchang had the lowest rate at 43.57%. However, there was no significant difference in the positive infection rate between different regions (*p* > 0.05) as shown in Fig. 2. Other factors such as gender, ethnicity, some lifestyle habits such as drinking raw water, washing hands before and after meals, location and frequency of eating out, and history of gastrointestinal symptoms or diseases were found to have no significant effect on *Hp* infection (Table 1).

**Table 1:** Prevalence of *Helicobacter pylori* infection and general information of the individual

Characteristics	All N(%)	*Hp* infected persons N(%)	*Hp* non-infected persons N(%)	Univariate analysis		Multivariable	
				OR(95%CI)	*p* value	OR(95%CI)	*p* value
Sex							
Male	606(44.72%)	269(44.39%)	337(55.61%)	Reference			
Female	749(55.28%)	360(48.06%)	389(51.94%)	1.16(0.94-1.44)	0.178		
Age							
0-9	137(10.11%)	29(21.17%)	108(78.83%)	Reference		Reference	
10-19	105(7.75%)	33(31.43%)	72(68.57%)	1.71(0.95-3.05)	0.071	1.50(0.78-2.88)	0.224
20-29	176(12.99%)	83(47.16%)	93(52.84%)	3.32(2.00-5.51)	0.000	2.66(1.27-5.58)	0.010
30-39	316(23.32%)	176(55.70%)	140(44.30%)	4.68(2.94-7.46)	0.000	3.70(1.66-8.23)	0.001
40-49	232(17.12%)	111(47.84%)	121(52.16%)	3.42(2.11-5.54)	0.000	2.59(1.16-5.76)	0.020
50-59	224(16.53%)	102(45.54%)	122(54.46%)	3.11(1.91-5.07)	0.000	2.38(1.09-5.21)	0.029
≥60	165(12.18%)	95(57.58%)	70(42.42%)	5.05(3.03-8.44)	0.000	4.01(1.83-8.79)	0.001
Living area							
City	846(62.44%)	395(46.69%)	451(53.31%)	Reference			
Urban-rural combination	374(27.60%)	170(45.45%)	204(54.55%)	0.95(0.75-1.21)	0.690		
Rural	135(9.96%)	64(47.41%)	71(52.59%)	1.03(0.72-1.48)	0.877		
Geographical location							
Haikou	502(37.05%)	231(46.02%)	271(53.98%)	Reference			
Danzhou	257(18.97%)	134(52.14%)	123(47.86%)	1.28(0.95-1.73)	0.110		
Wenchang	140(10.33%)	61(43.57%)	79(56.43%)	0.91(0.62-1.32)	0.608		
Dongfang	380(28.04%)	168(44.21%)	212(55.79%)	0.93(0.71-1.22)	0.594		
Changjiang Li Autonomous County	76(5.61%)	35(46.05%)	41(53.95%)	1.00(0.62-1.62)	0.995		
Ethnicity							
Han	928(87.46%)	422(45.47%)	506(54.53%)	Reference			
Li	63(5.94%)	33(52.38%)	30(47.62%)	1.32(0.79-2.20)	0.288		
Educational level							
Primary school or lower	292(21.55%)	98(33.56%)	194(66.44%)	Reference		Reference	
Junior or senior high school	371(27.38%)	186(50.13%)	185(49.87%)	0.51(0.38-0.68)	0.000	1.27(0.85-1.89)	0.251
College education or beyond	692(51.07%)	345(49.86%)	347(50.14%)	1.01(0.79-1.30)	0.931	1.03(0.64-1.66)	0.916
Educational level (exclusion adjustment for the age variable in the multivariate regression analysis)							
Primary school or lower	292(21.55%)	98(33.56%)	194(66.44%)	Reference		Reference	
Junior or senior high school	371(27.38%)	186(50.13%)	185(49.87%)	0.51(0.38-0.68)	0.000	1.64(1.18-2.29)	0.004
College education or beyond	692(51.07%)	345(49.86%)	347(50.14%)	1.01(0.79-1.30)	0.931	1.65(1.22-2.22)	0.001
Marital status							
Unmarried	384(28.34%)	135(35.16%)	249(64.84%)	Reference		Reference	
Married	954(70.41%)	486(50.94%)	468(49.06%)	1.92(1.50-2.45)	0.000	0.89(0.58-1.37)	0.591
Other	17(1.25%)	8(47.06%)	9(52.94%)	1.64(0.62-4.35)			
Marital status (exclusion adjustment for the age variable in the multivariate regression analysis)							
Unmarried	384(28.34%)	135(35.16%)	249(64.84%)	Reference		Reference	
Married	954(70.41%)	486(50.94%)	468(49.06%)	1.92(1.50-2.45)	0.000	1.64(1.27-2.13)	0.000
Other	17(1.25%)	8(47.06%)	9(52.94%)	1.64(0.62-4.35)	0.32	1.53(0.57-4.09)	0.395
Drink raw water							
No	1311(96.75%)	612(46.68%)	699(53.32%)	Reference	0.295		
Yes	44(3.25%)	17(38.64%)	27(61.36%)	0.72(0.32-1.33)			
Wash hands before meals and after defecation							
No	110(8.12%)	58(52.73%)	52(47.27%)	Reference			
Yes	1245(91.88%)	571(45.86%)	729(58.55%)	0.76(0.51-1.12)	0.168		
Dining in the school/Unit Canteen							
Rarely	1263(93.21%)	586(46.40%)	677(53.60%)	Reference			
More than two days per week	92(6.79%)	43(46.74%)	49(53.26%)	1.01(0.66-1.55)	0.949		
Dining in the small restaurants							
Rarely	1133(83.62%)	525(46.34%)	608(53.66%)	Reference			
More than two days per week	222(16.38%)	104(46.85%)	118(53.15%)	1.02(0.77-1.36)	0.889		
Dining in the hotel restaurant							
Rarely	1251(92.32%)	581(46.44%)	670(53.56%)	Reference			
More than two days per week	104(7.68%)	48(46.15%)	56(53.85%)	0.99(0.66-1.48)	0.955		
Gastrointestinal discomfort within the last 1 year							
No	912(67.31%)	426(46.71%)	486(53.29%)	Reference			
Yes	443(32.69%)	203(45.82%)	240(54.18%)	0.96(0.77-1.21)	0.759		
Gastroscopy within 5 years							
No	1160(85.61%)	549(47.33%)	611(52.67%)	Reference			
Yes	195(14.39%)	80(41.03%)	115(58.97%)	0.77(0.57-1.05)	0.103		
Gastroscopic diagnosis							
Chronic gastritis	105(7.75%)	46(43.81%)	59(56.19%)	Reference			
Peptic ulcer	31(2.29%)	12(38.71%)	19(61.29%)	0.81(0.36-1.84)	0.614		
Others	29(2.14%)	10(34.48%)	19(65.52%)	0.68(0.29-1.59)	0.369		
Unknown	30(2.21%)	12(40.00%)	18(60.00%)	0.86(0.37-1.95)	0.710		
Previously tested for *Hp* infection							
No	1125(83.03%)	530(47.11%)	595(52.89%)	Reference			
Yes	230(16.97%)	99(43.04%)	131(56.96%)	0.85(0.64-1.13)	0.260		
Previous anti-*Hp* treatment							
No	1254(92.55%)	588(46.89%)	666(53.11%)	Reference			
Yes	101(7.45%)	41(40.59%)	60(59.41%)	0.77(0.51-1.77)	0.223		
History of gastroduodenal surgery							
No	1343(99.11%)	4(33.33%)	8(66.67%)	Reference			
Yes	12(0.89%)	4(33.33%)	8(66.67%)	0.57(0.17-1.92)	0.367		
Total	1355	629(46.42%)	726(53.58%)				

*Hp* *Helicobacter pylori*, *OR* Odds Ratio, *CI* Confidence interval

### *Helicobacter pylori* infection in households

**Fig. 3:**
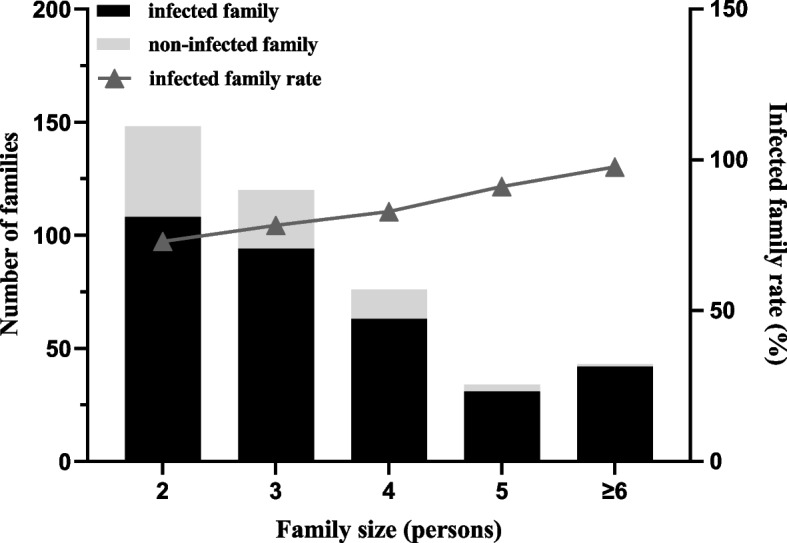
The general status of *Helicobacter pylori* infection in the family Note: Infected family: At least one family member infected with *Helicobacter pylori*

Out of the 1355 people, there were a total of 421 households with more than 2 members per household. Among these households, 80.29% (338/421) had at least 1 family member infected with *Hp*, while 19.71% (83/421) had no household members infected with *Hp*. Additionally, the infection rate tended to increase as the size of the household increased (Fig. 3).

**Fig. 4:**
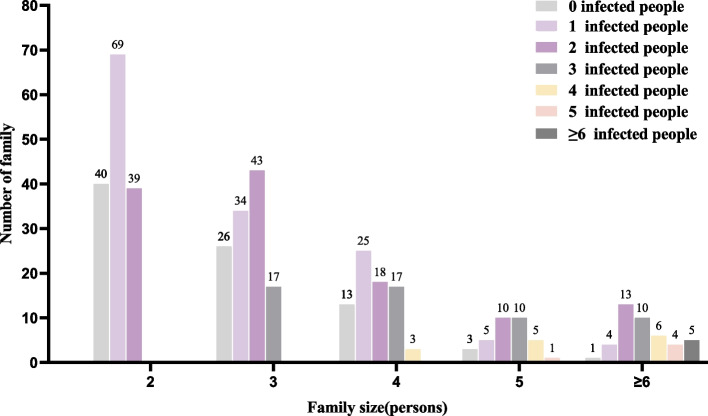
Distribution of *Helicobacter pylori* infection in the household

In households infected with *Hp*, more than half of them consisted of 2–3 persons. Among all infected households, 1 and 2 persons were the most commonly infected with *Hp*, accounting for 40.53% and 36.39% respectively. When the household size was 5 or more people, 2–3 people were more likely to be concurrently infected with *Hp* (Table 2). Figure 4 displayed the distribution of *Hp*-uninfected households and *Hp*-infected persons, indicating that larger households had a lower distribution of *Hp*-uninfected individuals.

**Table 2:** Distribution of *Helicobacter pylori* infection among family members

Family size (persons)	Total N (%)	1 infected person N (%)	2 infected persons N (%)	3 infected persons N (%)	4 infected persons N (%)	5 infected persons N (%)	≥ 6 infected persons N (%)
2	108(31.95%)	69(63.89%)	39(36.11%)				
3	94(27.81%)	34(36.17%)	43(45.74%)	17(18.09%)			
4	63(18.64%)	25(39.68%)	18(28.57%)	17(26.98%)	3(4.76%)		
5	31(9.17%)	5(16.13%)	10(32.26%)	10(32.26%)	5(16.13%)	1(3.23%)	
≥ 6	42(12.43%)	4(9.52%)	13(30.95%)	10(23.81%)	6(14.29%)	4(9.52%)	5(11.90%)
Total N (%)	338(100%)	137(40.53%)	123(36.39%)	54(15.98%)	14(4.14%)	5(1.48%)	5(1.48%)

The risk of *Hp* infection was higher in households with 5, 6, or more persons, with ORs of 4.09 (1.17–14.33) and 15.19 (2.01–114.73), respectively. Conversely, households with an income of ≥ 100,000 yuan and those that drank boiled water from tap sources were found to be protective factors against *Hp* infection, with ORs of 0.52 (0.31–0.89) and 0.51 (0.28–0.95), respectively. Other factors such as living area, family history of disease, domestic animals, and family habits such as meal gatherings, use of communal chopsticks, and sharing of objects did not have a significant effect on household *Hp* infection (Table 3).

**Table 3:** Prevalence of *Helicobacter pylori* infection and general information of the family

Characteristics	Total families N(%)	*Hp* infected families N(%)	*Hp* non-infected families N(%)	Univariate analysis		Multivariable analysis	
				OR(95%CI)	*p* value	OR(95%CI)	*p* value
Family size (persons)							
2	148(35.15%)	108(72.97%)	40(27.03%)	Reference		Reference	
3	120(28.50%)	94(78.33%)	26(21.67%)	1.34(0.76-2.36)	0.312	1.30(0.73-2.32)	0.374
4	76(18.05%)	63(82.89%)	13(17.11%)	1.79(0.89-3.61)	0.101	1.68(0.82-3.43)	0.155
5	34(8.08%)	31(91.18%)	3(8.82%)	3.83(1.11-13.22)	0.034	4.09(1.17-14.33)	0.028
≥6	43(10.21%)	42(97.67%)	1(2.33%)	15.56(2.06-116.80)	0.008	15.19(2.01-114.73)	0.008
The generations living together							
1	85(20.19%)	63(74.12%)	22(25.88%)	Reference			
2	214(50.83%)	175(81.78%)	39(18.22%)	1.57(0.86-2.85)	0.140		
≥3	122(28.98%)	100(81.97%)	22(18.03%)	1.59(0.81-3.10)	0.176		
Family income (yuan)							
＜100,000	300(71.26%)	249(83.00%)	51(17.00%)	Reference		Reference	
≥100,000	121(28.74%)	89(73.55%)	32(26.45%)	0.57(0.34-0.94)	0.029	0.52(0.31-0.89)	0.017
Living area							
City	268(63.66%)	209(77.99%)	59(22.01%)	Reference			
Urban-rural combination	111(26.37%)	93(83.78%)	18(16.22%)	1.46(0.82-2.61)	0.203		
Rural	42(9.98%)	36(85.71%)	6(14.29%)	1.69(0.68-4.21)	0.257		
Total household living area (m^2^)							
＜60	51(12.11%)	42(82.35%)	9(17.65%)	Reference			
60-120	263(62.47%)	210(79.85%)	53(20.15%)	0.85(0.39-1.85)	0.681		
＞120	107(25.42%)	86(80.37%)	21(19.63%)	0.88(0.37-2.08)	0.767		
Family with animals							
No	363(86.22%)	288(79.34%)	75(20.66%)	Reference			
Yes	58(13.78%)	50(86.21%)	8(13.79%)	1.63(0.74-3.58)	0.226		
Family with Pets							
No	397(94.30%)	316(79.60%)	81(20.40%)	Reference			
Yes	24(5.70%)	22(91.67%)	2(8.33%)	2.82(0.65-12.24)	0.166		
Family with Poultry							
No	387(91.92%)	309(79.84%)	78(20.16%)	Reference			
Yes	34(8.08%)	29(85.29%)	5(14.71%)	1.46(0.55-3.91)	0.446		
Family with Livestock							
No	409(97.15%)	329(80.44%)	80(19.56%)	Reference			
Yes	12(2.85%)	9(75.00%)	3(25.00%)	0.73(0.19-2.76)	0.642		
Drinking water sources							
Heated tap water	338(80.29%)	278(82.25%)	60(17.75%)	Reference		Reference	
Raw tap water	67(15.91%)	47(70.15%)	20(29.85%)	0.51(0.28-0.92)	0.025	0.51(0.28-0.95)	0.033
Bottled water	4(0.95%)	3(75.00%)	1(25.00%)	0.65(0.07-6.33)	0.709	0.79(0.08-8.09)	0.840
Well water	10(2.38%)	9(90.00%)	1(10.00%)	1.94(0.24-15.62)	0.532	2.23(0.27-18.27)	0.454
Others	2(0.48%)	1(50.00%)	1(50.00%)	0.22(0.01-3.50)	0.281	0.28(0.02-5.16)	0.391
Dish scrubbing							
Flow washing	359(85.27%)	286(79.67%)	73(20.33%)	Reference			
Still water / basin wash	62(14.73%)	52(83.87%)	10(16.13%)	1.33(0.64-2.74)	0.443		
Dish sterilization							
No sterilization	162(38.48%)	127(78.40%)	35(21.60%)	Reference			
Automatic sterilizer	191(45.37%)	157(82.20%)	34(17.80%)	1.27(0.75-2.15)	0.370		
Other disinfection methods	68(16.15%)	54(79.41%)	14(20.59%)	1.06(0.53-2.13)	0.864		
Sharing of household goods							
No	143(33.97%)	115(80.42%)	28(19.58%)	Reference			
Yes	278(66.03%)	223(80.22%)	55(19.78%)	0.99(0.59-1.64)	0.960		
Dishes and Chopsticks sharing							
No	157(37.29%)	127(80.89%)	30(19.11%)	Reference			
Yes	264(62.71%)	211(79.92%)	53(20.08%)	0.94(0.57-1.55)	0.809		
Tea cup sharing							
No	350(83.14%)	282(80.57%)	68(19.43%)	Reference			
Yes	71(16.86%)	56(78.87%)	15(21.13%)	0.90(0.48-1.69)	0.743		
Mouthwash Cup sharing							
No	374(88.84%)	297(79.41%)	77(20.59%)	Reference			
Yes	47(11.16%)	41(87.23%)	6(12.77%)	1.77(0.73-4.33)	0.209		
Tooth cleaner sharing							
No	401(95.25%)	321(80.05%)	80(19.95%)	Reference			
Yes	20(4.75%)	17(85.00%)	3(15.00%)	1.41(0.40-4.94)	0.589		
Family meal sharing							
No	343(81.47%)	277(80.76%)	66(19.24%)	Reference			
Yes	78(18.53%)	61(78.21%)	17(21.79%)	0.85(0.47-1.56)	0.609		
Use communal chopsticks and spoons							
No	332(78.86%)	270(81.33%)	62(18.67%)	Reference			
Yes	89(21.14%)	68(76.40%)	21(23.60%)	0.74(0.42-1.30)	0.301		
Family history of peptic ulcer							
No	328(77.91%)	267(81.40%)	61(18.60%)	Reference			
Yes	93(22.09%)	71(76.34%)	22(23.66%)	0.74(0.42-1.28)	0.280		
Family history of gastric cancer							
No	410(97.39%)	327(79.76%)	83(20.24%)	Reference			
Yes	11(2.61%)	11(100.00%)	0(0.00%)	0.80(0.76-0.84)	0.200		
Health workers in the home							
No	207(49.17%)	161(77.78%)	46(22.22%)	Reference			
Yes	214(50.83%)	177(82.71%)	37(17.29%)	1.37(0.84-2.21)	0.204		
Male to female ratio in the household	0.44±0.21	0.44±0.21	0.44±0.21		0.575		
Average age of family members	38.22±10.52	38.69±10.32	36.29±11.13		0.141		
Percentage of family members tested for *Hp*^a^	0.19±0.29	0.19±0.29	0.18±0.28		0.908		
Percentage of family members with higher education^b^	0.54±0.33	0.54±0.32	0.55±0.37		0.085		
Duration of family cohabitation (years)							
＜1	41(9.74%)	34(82.93%)	7(17.07%)	Reference			
≥1	88(20.90%)	70(79.55%)	18(20.45%)	0.80(0.31-2.10)	0.651		
≥5	118(28.03%)	99(83.90%)	19(16.10%)	1.07(0.41-2.77)	0.885		
≥10	174(41.33%)	135(77.59%)	39(22.41%)	0.71(0.29-1.73)	0.455		
At least one person infected *Hp*							
No	83(19.71%)						
Yes	338(80.29%)						

*Hp Helicobacter pylori*, *OR* Odds Ratio, *CI* Confidence interval

### *Helicobacter pylori* infection in minors

**Fig. 5:**
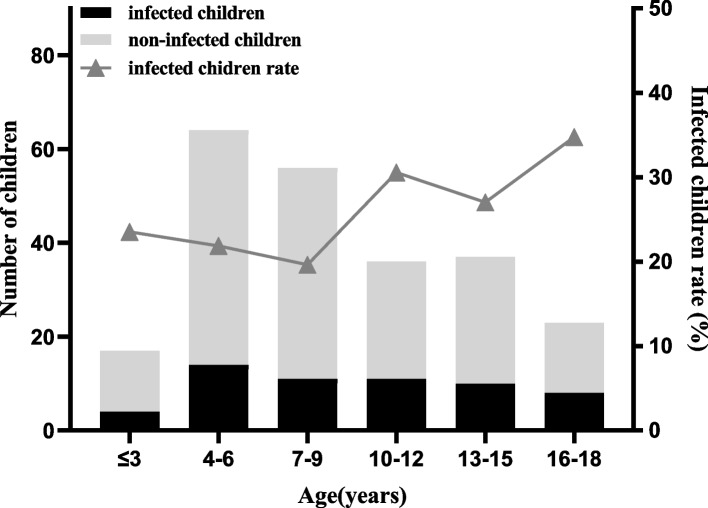
Helicobacter pylori infection status in children of different age groups

A total of 233 minors, consisting of 119 boys and 114 girls, were included in the study. Of these minors, 24.89% (58/233) were infected with *Hp* and 75.11% (175/233) were not infected. The minors were divided into six age groups, with the highest infection rate of 34.78% (8/37) occurring in the 16–18 years old group. As shown in Fig. 5, there was a tendency for the infection rate to increase with age. However, the rate of *Hp* positivity did not show a significant difference among the age groups (*p* > 0.05).

In the study examining the factors contributing to *Hp* infection in minors, it was found that father's infection and mother's infection were risk factors for children's infection, with ORs of 2.93 (1.29–6.62) and 2.51 (1.07–5.89) respectively in the multivariable logistic regression analysis. Lifestyle habits such as drinking raw water, washing hands before and after meals, and close interaction with parents (including mouth-to-mouth kissing) did not have a significant effect on *Hp* infection in minors, even when infected by other elders (Table 4).

**Table 4:** Prevalence of *Helicobacter pylori* infection and the lifestyle of children in the family

Characteristics	All N(%)	*Hp* infected persons N(%)	*Hp* non-infected persons N(%)	Univariate analysis		Multivariable analysis	
				OR(95%CI)	*p* value	OR(95%CI)	*p* value
Total	233	58(24.89%)	175(75.11%)				
Sex							
boy	119(51.07%)	29(24.37%)	90(75.63%)	Reference			
girl	114(48.93%)	29(25.44%)	85(74.56%)	1.06(0.58-1.92)	0.850		
Age(years)							
≤3	17(7.30%)	4(23.53%)	13(76.47%)	Reference			
4-6	64(27.47%)	14(21.88%)	50(78.13%)	0.91(0.26-3.23)	0.884		
7-9	40(17.17%)	11(19.64%)	45(80.36%)	0.94(0.25-3.62)	0.932		
10-12	40(17.17%)	11(30.56%)	25(69.44%)	1.08(0.29-4.09)	0.906		
13-15	35(15.02%)	10(27.03%)	27(72.97%)	1.30(0.34-4.96)	0.701		
16-18	37(15.88%)	8(34.78%)	15(65.22%)	1.37(0.37-5.17)	0.637		
Drink raw water							
No	221(94.85%)	55(24.89%)	166(75.11%)	Reference			
Yes	12(5.15%)	3(25.00%)	9(75.00%)	1.01(0.26-3.85)	0.993		
Wash hands before meals and after defecation							
No	19(8.15%)	7(36.84%)	12(63.16%)	Reference			
Yes	214(91.85%)	51(23.83%)	163(76.17%)	0.54(0.20-1.43)	0.215		
Dining in the school/Unit Canteen							
Rarely	223(95.71%)	55(24.66%)	168(75.34%)	Reference			
More than two days per week	10(4.29%)	3(30.00%)	7(70.00%)	1.31(0.33-5.24)	0.703		
Dining in the small restaurants							
Rarely	219(93.99%)	54(24.66%)	165(75.34%)	Reference			
More than two days per week	14(6.01%)	4(28.57%)	10(71.43%)	1.22(0.37-4.06)	0.743		
Dining in the hotel restaurant							
Rarely	225(96.57%)	57(25.33%)	168(74.67%)	Reference			
More than two days per week	8(3.43%)	1(12.50%)	7(87.50%)	0.423(0.05-3.50)	0.423		
Children's habit of sticking toys or objects in their mouths							
No	177(91.71%)	44(24.86%)	133(75.14%)	Reference			
Yes	16(8.29%)	2(12.50%)	14(87.50%)	0.43(0.09-1.97)	0.279		
Parents often kiss their children mouth-to-mouth							
No	170(88.08%)	40(23.53%)	130(76.47%)	Reference			
Yes	23(11.92%)	6(26.09%)	17(73.91%)	1.15(0.42-3.11)	0.787		
children entering kindergarten							
No	59(30.57%)	16(27.12%)	43(72.88%)	Reference			
Yes	134(69.43%)	30(22.39%)	104(77.61%)	0.78(0.38-1.57)	0.478		
Mother infected							
No	86(45.50)	14(16.28%)	72(83.72%)	Reference		Reference	
Yes	103(54.50%)	35(33.98%)	68(66.02%)	2.65(1.31-5.35)	0.007	2.51(1.07-5.89)	0.035
Father infected							
No	99(60.37%)	14(14.14%)	85(85.86%)	Reference		Reference	
Yes	65(39.63%)	23(35.38%)	42(64.62%)	3.33(1.55-7.11)	0.002	2.93(1.29-6.62)	0.010
Grandmother infected							
No	38(43.18%)	4(10.53%)	34(89.47%)	Reference			
Yes	50(56.82%)	9(18.00%)	41(82.00%)	1.87(0.53-6.59)	0.333		
Grandfather infected							
No	22(41.51%)	2(9.09%)	20(90.91%)	Reference			
Yes	31(58.49%)	6(19.35%)	25(80.65%)	2.40(0.44-13.20)	0.314		

*Hp Helicobacter pylori*, *OR* Odds Ratio, *CI* Confidence interval

## Discussion

According to recent studies, approximately 589 million people in mainland China have been infected with *Hp*. While the rate of infection has decreased compared to 20 years ago, the burden of infection remains high. While the traditional "screen and treat" approach has been recommended for treating individuals infected *Hp*, in 2021 China proposed a new family unit-based treatment strategy to prevent further transmission of *Hp* among family members by systematically following up and screening for the *Hp* status of other family members of those infected with *Hp*, as well as evaluating and treating them [8]. On top of this, exploring potential modes of *Helicobacter pylori* transmission within families can aid in mitigating the risk of both initial infection and reinfection.

This study investigated the *Hp* infection status of 1355 individuals from 421 households on Hainan Island and analyzed the factors influencing *Hp* infection using a questionnaire. The findings revealed that 80.29% (338/421) of households had at least one person infected with *Hp*. These results suggested that a significant proportion of households on Hainan Island were at risk of *Hp* infection. In the analysis of factors influencing household *Hp* infection, it was found that family size of more than five people was a risk factor. Since each person in the household was susceptible to *Hp*, having more people in the household meant more individuals were at risk and therefore increased the likelihood of exposure to *Hp* infection. In contrast, high income (≥ 100,000 yuan) and consumption of boiled tap water were found to be protective factors against the transmission of *Hp*. This finding was consistent with previous studies that have identified factors influencing *Hp* transmission [9, 10]. *Hp* can be transmitted through various water sources, including bottled water, tap water, and well water. Additionally, *Hp* can adhere to different materials and coexist with other bacteria in pipes and on water surfaces [11, 12]. *Hp* can withstand challenging conditions such as micro-oxygenation and pH levels ranging from 4.5 to 9.0. It can even survive for up to two weeks at a temperature of 4°C [13, 14]. The prevalence of *Hp* in drinking water was high, with a global average of 15.7%, and drinking such water can lead to *Hp* infection [15]. To prevent this, it is recommended to follow good hygiene practices, such as drinking boiled and professionally disinfected tap water.

In addition to external factors, such as contaminated water or food, that can lead to family-based *Hp* infection, it is important to also consider the oral-oral route of transmission. This survey found no correlation between household members' use of public utensils and household products and *Hp* infection. It is important to note that *Hp* must pass through the mouth before colonizing the stomach and can be detected in various oral sources such as plaque, saliva, tongue, and dental pulp [16]. The oral cavity, as the first reservoir of *Hp* outside the stomach, was closely related to *Hp* infection in the stomach [17]. In China, it is common for family members to use their own cutlery, such as chopsticks and spoons, to obtain and share food during meals. However, an Australian study has shown that the use of chopsticks may promote *Hp* infection due to the possibility of cross-contamination during this process [18]. However, this study on *Hp* detection was based on a serological test and did not directly prove the presence of these microorganisms on the chopsticks. A subsequent trial in Hong Kong detected *Hp* by PCR in the saliva of 15 (33%) *Hp*-infected subjects and in the chopsticks of one (2%), indicating a high likelihood of direct transmission of the bacteria through infected saliva. Although the detection rate of *Hp* on chopsticks is low, it is still infectious, and communal utensils should be used with caution during meals [19].

This study also analyzed *Hp* infection on an individual unit basis and the factors influencing *Hp* infection. The results showed that 46.72% of the participants were infected with *Hp*, which is consistent with the medium prevalence of *Hp* in Hainan Province, relative to other provinces in China. The study investigated the prevalence of *Hp*-positive infections across different age groups. The results showed that the prevalence increased with age, particularly in the 0–40 age group, which is consistent with previous studies [10, 20]. It seemed that the risk of *Hp* infection in the population was concentrated in children and young adults, thus, the study focused on analyzing *Hp* infection in minors.

The survey found that the prevalence of *Hp* infection among infants and children aged 0–3 years was as high as 23.54%. The prevalence of infection varied between countries and regions. Studies in Norway showed almost no infection in children aged 0–11 years (0.6%), while Turkey had 10.71% infection in children aged 0–5 years. In Wuwei, Gansu Province, China, 12.6% of infants aged 0–3 years had infection, while in Hong Kong, 9.3% of children aged 6–9 years were infected [10, 21–23]. The results suggested a correlation between the economic status of an area and the prevalence of *Hp* infection in children, with more economically developed areas having lower rates of infection. However, further validation through normative studies with larger samples and a wider range of areas is necessary due to variations in sample size, testing protocols, and survey time across studies. This study confirmed that the majority of *Hp* infections occurred during early childhood, as evidenced by the high prevalence of infection among children aged 0–3 years. A previous study of 231 Israeli children found that *Hp* infection typically occurred around 14 months of age, and identified low income, low-education parents and poor hygiene practices (such as infrequent sterilization of bottles and teats) as important risk factors for *Hp* infection during infancy [24]. Breastfeeding has been found to reduce the risk of *Hp* infection in infants [25]. Additionally, a study has shown that specific immunoglobulin A antibodies found in breast milk can delay *Hp* fixation in infants [26]. However, children attending day-care facilities have been found to be at an increased risk of infection. The risk of infection was observed to increase significantly with the cumulative time spent in day-care centers (*p* < 0.001) [27].

The study found that the prevalence of *Hp* infection increased with the age of the child, particularly in those who were 10 years or older, with rates exceeding 25%. The risk of infection in children was also found to be higher when family members, especially fathers (OR: 2.93, 95% CI: 1.29–6.62) and mothers (OR: 2.51, 95% CI: 1.07–5.89), were infected, which was in line with previous cross-sectional studies [28, 29]. Most studies have confirmed that parents play an important role in the transmission of Hp within the family, especially as mothers, whether based on genetic analysis or ^13^C-UBT [30–32]. Studies from Germany showed that paternal Hp infection was a risk factor with a crude OR of 7.8 (95% CI, 2.5–24.2) and an OR of 3.8 (95% CI, 0.8–19.1) after adjusting for potential confounders (except maternal infection) [33]. This indicates that the role of fathers in spreading hp within the family cannot be ignored. At the same time, the longer the exposure time of HP-positive parents caring for their children, the greater the risk of infection of the child. In this study, fathers had a more significant OR value, and it cannot be ruled out that fathers were the main caregivers in the convenient sample population for this study.

A study conducted in Japan found that the strains of *Hp* isolated from children were similar to those of their siblings [34]. Similarly, a Swedish study that compared strains isolated from *Hp*-infected children aged 10–12 years in school and their infected family members arrived at similar conclusions [4]. The transmission of *Hp* was found to be stronger from mother to child than between father and son or siblings. This mode of transmission played a key role in the spread of *Hp *[35]. According to data released by the Hainan Provincial Bureau of Statistics in 2021, the average number of persons per household in Hainan Province decreased from 4.86 in 1982 to 3.06 in 2021. Due to the one child per household family planning policy encouraged from 2002 to 2016, it was even less likely that most families had children with *Hp* infection had originated from their siblings [36].

The survey also indicated that individuals with education beyond junior high school and those who were married were at higher risk of *Hp* infection. The transmission of the infection between spouses was also a significant factor to consider, in addition to the transmission from parents to children. A German survey found that the risk of *Hp* infection increased with the number of years an individual lives with an infected partner, indicating that *Hp* can be transmitted between spouses [37]. The Swedish study used methods such as random amplified polymorphic DNA markers to validate isolated strains in 23 couples who were both infected with *Hp*. The results revealed that five of the couples shared the same strain, thereby indicating the likelihood of inter-spousal transmission of *Hp *[4].

The prevalence of *Hp* infection in Hainan was still significant, particularly within households. This study served as a valuable resource for identifying the primary sources of household-based *Hp* infection and its transmission in Hainan. These findings reinforced the need for a family-based approach to *Hp* eradication treatment. The use of convenience sampling to obtain the sample for this study may have introduced bias in the selection of the population. However, the large sample size likely mitigated this effect. It is important to note that this was a cross-sectional survey study, and as such, the population was not followed up with a before-and-after control to establish causality. Further research is needed to strengthen the conclusion of this study.

### Supplementary Information

**Additional file 1.**

## Data Availability

Data for this study can be obtained by contacting the corresponding author by email.

## Abbreviations

*Hp*: *Helicobacter pylori*
OR: Odds Ratio
^13^C-UBT: ^13^C-Urea Breath Test
CI: Confidence Interval
SAT: Stool Antigen Test
