# Supplementary material for: A survey on the current status of Helicobacter pylori infection in households in Hainan Province, China
Source: BMC Gastroenterol. 2023 Dec 4;23:426. doi: 10.1186/s12876-023-03010-z (PMC10696850; doi:10.1186/s12876-023-03010-z)
Supplement: Supplementary file 1 — Additional file 1. [file 12876_2023_3010_MOESM1_ESM.docx]

**Questionnaire**

| Family ID |  | Name of household head |  | Phone number |  | Family size |  | |
| --- | --- | --- | --- | --- | --- | --- | --- | --- |
| Family composition | 1. Head of the household; 2. Husband; 3. Wife; 4. Children; 5. Father; 6. Mother; 7. Brothers and sisters; 8. Daughter-in-law/Son-in-law; 9. Grandfather; 10. Grandmother; 11. Grandchildren; 12. Granddaughters-in-law/Son-in-law; 13. Others | | | | | | | |
| Family address | Province City District Street Road | | | | | | | |
| **Question for the family** | | | | | | | | |
| **1. Family economy and living conditions** | | | | | | | | |
| 1.1 | Annual family income (yuan): 1. ＜100,000; 2. ≥100,000 | | | | | | |  |
| 1.2 | Living area: 1. City; 2. Urban-rural combination; 3. Rural areas | | | | | | |  |
| 1.3 | Total family living area (m^2^): 1. <60; 2.60-120; 3.> 120 | | | | | | |  |
| 1.4 | Do you have any animals at home? 1. No; 2. Yes | | | | | | |  |
| 1.4.1 | If yes, what kind of animal is it? 1. Pet; 2. Poultry; 3. Livestock | | | | | | |  |
| **2. Family hygiene and living habits** | | | | | | | | |
| 2.1 | Household drinking water mainly comes from: 1. Heated tap water; 2. Raw tap water; 3. Bottled water; 4. Well water; 5. Other | | | | | | |  |
| 2.2 | How is the household dish washing? 1. Flow washing; 2. Still water/basin washing | | | | | | |  |
| 2.3 | How about the disinfection of household dishes and chopsticks? 1. No disinfection; 2. Automatic disinfection cabinet; 3. Other disinfection methods | | | | | | |  |
| 2.4 | Whether there are the following items shared in the family? 1. Bowls and chopsticks; 2. Tea cups; 3. Mouthwash cups; 4. Tooth cleaner | | | | | | |  |
| 2.5 | Do you use separate meals for family meals? 1. Yes; 2. No | | | | | | |  |
| 2.6 | Do you use communal chopsticks and spoons for family meals? 1. Yes; 2. No | | | | | | |  |
| **3. Family history of disease (including deceased members within three generations)** | | | | | | | | |
| 3.1 | Whether there are the following diseases? 1. Peptic ulcer; 2. Gastric cancer; 3. No | | | | | | |  |

**Question for individual family members (each member fills in the following questions)**

| **4. General information of family members** | | | | | | | | |
| --- | --- | --- | --- | --- | --- | --- | --- | --- |
| Family ID |  | Name |  | Contact number |  | ID number |  | |
| Gender | 1.Male； 2. Female | Ethnicity |  | date of birth |     Year   Month   Day | | | |
| 4.1 | Time spent together in the family: 1. <1 year；2. ≥1 year； 3. ≥5 years； 4. ≥10 years | | | | | | |  |
| 4.2 | Marital status: 1 unmarried; 2 married; 3. Other | | | | | | |  |
| 4.3 | Education level: 1. Primary school or below; 2. Junior or senior high school; 3. College education or beyond | | | | | | |  |
| **5. Personal hygiene and lifestyle habits** | | | | | | | | |
| 5.1 | Do you drink raw water?1. No; 2. Yes | | | | | | |  |
| 5.2 | Do you wash your hands before eating and after using the toilet?1. No; 2. Yes | | | | | | |  |
| 5.3 | Frequency of eating out: 1. Few; 2. More than two days a week | | | | | | |  |
| 5.3.1 | If you choose the second option, please fill in the dining place: 1. School / unit canteen; 2. Small restaurant; 3. Hotel restaurant | | | | | | |  |
| **6. Previous medical history** | | | | | | | | |
| 6.1 | Whether there are gastrointestinal discomfort symptoms in the past 1 year: 1. No; 2. Yes | | | | | | |  |
| 6.2 | Have gastroscopy in 5 years: 1. No; 2. Yes | | | | | | |  |
| 6.2.1 | If so, gastroscopy diagnosis: 1. Chronic gastritis; 2. Peptic ulcer; 3. Gastric cancer; 4. Other; 5. Unknown | | | | | | |  |
| 6.3 | Have you ever been tested for Helicobacter pylori infection: 1. No; 2. Yes | | | | | | |  |
| 6.4 | Any anti-H. pylori treatment: 1. No; 2. Yes | | | | | | |  |
| 6.5 | History of gastroduodenal surgery; 1. No; 2. Yes | | | | | | |  |
| **7. The Hp results were detected in this survey** | | | | | | | | |
| 7.1 | Results: 1. Positive; 2. Negative | | | | | | |  |
| **8. Families with children aged 1-18 will continue to fill in the following questions** | | | | | | | | |
| 8.1 | Whether parents have the habit of chewing feeding food or feeding it mouth to mouth: 1. No; 2. Yes | | | | | | |  |
| 8.2 | Whether children have the habit of putting toys or objects into their mouth: 1. No; 2. Yes | | | | | | |  |
| 8.3 | Do parents often kiss their children from mouth to mouth: 1. No; 2. Yes | | | | | | |  |
| 8.4 | Kindergarten attendance: 1. No; 2. Yes | | | | | | |  |

Investigator (signature): Date of investigation：    Year   Month   Day
